# Bacterial division ring stabilizing ZapA versus destabilizing SlmA modulate FtsZ switching between biomolecular condensates and polymers

**DOI:** 10.1098/rsob.220324

**Published:** 2023-03-01

**Authors:** Begoña Monterroso, Miguel Ángel Robles-Ramos, Marta Sobrinos-Sanguino, Juan Román Luque-Ortega, Carlos Alfonso, William Margolin, Germán Rivas, Silvia Zorrilla

**Affiliations:** ^1^ Department of Structural and Chemical Biology, Centro de Investigaciones Biológicas Margarita Salas, Consejo Superior de Investigaciones Científicas (CSIC), 28040 Madrid, Spain; ^2^ Molecular Interactions Facility, Centro de Investigaciones Biológicas Margarita Salas, Consejo Superior de Investigaciones Científicas (CSIC), 28040 Madrid, Spain; ^3^ Department of Microbiology and Molecular Genetics, McGovern Medical School, UTHealth-Houston, Houston, TX 77030, USA

**Keywords:** biomolecular condensates, bacterial division, crowding-driven phase separation, membraneless compartments, subcellular organization, biochemical reconstitution in cytomimetic media

## Abstract

Cytokinesis is a fundamental process for bacterial survival and proliferation, involving the formation of a ring by filaments of the GTPase FtsZ, spatio-temporally regulated through the coordinated action of several factors. The mechanisms of this regulation remain largely unsolved, but the inhibition of FtsZ polymerization by the nucleoid occlusion factor SlmA and filament stabilization by the widely conserved cross-linking protein ZapA are known to play key roles. It was recently described that FtsZ, SlmA and its target DNA sequences (SlmA-binding sequence (SBS)) form phase-separated biomolecular condensates, a type of structure associated with cellular compartmentalization and resistance to stress. Using biochemical reconstitution and orthogonal biophysical approaches, we show that FtsZ-SlmA-SBS condensates captured ZapA in crowding conditions and when encapsulated inside cell-like microfluidics microdroplets. We found that, through non-competitive binding, the nucleotide-dependent FtsZ condensate/polymer interconversion was regulated by the ZapA/SlmA ratio. This suggests a highly concentration-responsive tuning of the interconversion that favours FtsZ polymer stabilization by ZapA under conditions mimicking intracellular crowding. These results highlight the importance of biomolecular condensates as concentration hubs for bacterial division factors, which can provide clues to their role in cell function and bacterial survival of stress conditions, such as those generated by antibiotic treatment.

## Graphical abstract

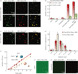

## 1. Background

Bacterial cell division is achieved through the formation of a protein ring at midcell, its scaffold being the GTPase self-assembling protein FtsZ [1]. The coalescence of FtsZ into a ring-like structure is one of the first events of division in most bacteria [2,3], followed by the subsequent recruitment of over 30 proteins [2] to form the machinery that ultimately splits the mother cell into two daughter cells. The FtsZ ring is subjected to tight regulation to ensure its formation only at the cell centre towards the end of the cell cycle [4]. Most of the systems involved in the regulation of this cytoskeletal structure exert their action through direct or indirect interaction with FtsZ, ultimately modulating its assembly properties. In addition to GTP-induced polymers, which disassemble upon accumulation of GDP due to GTP hydrolysis by the protein [5], FtsZ also forms oligomers in the absence of GTP, and both association processes depend on salt and magnesium concentrations [5,6]. Interestingly, many regulators bind to polymers and oligomers of FtsZ, indicating that the two types of assemblies may be important for the fine tuning of FtsZ ring formation. The relevance of the FtsZ oligomers has been lately revealed as even wider by their ability to form biomolecular condensates in synthetic systems mimicking natural crowding [7,8]. As participants in such a fundamental process, division proteins are emerging targets of potential strategies to fight pernicious bacteria [9].

Regulatory systems are classified either as negatives that counteract FtsZ ring formation at wrong locations in the cell, or positives that act as stabilizers of ring assembly at the division site [10]. One of the most ubiquitous and widely studied positive regulators is ZapA. This protein is broadly conserved among Gram-negative and Gram-positive bacteria, and is known to stabilize FtsZ polymers and to promote bundling of FtsZ filaments *in vitro* [11,12]. Deletion of ZapA *in vivo* results in FtsZ rings consisting of smaller and more dispersed FtsZ clusters [13]. ZapA has been described to reverse the effects of MinC, a negative regulator that directly interacts with FtsZ as part of the oscillating Min system that selectively inhibits FtsZ ring assembly at cell poles [14]. Indeed, ZapA was discovered as a suppressor of excess levels of Min proteins in *B. subtilis* [15]. ZapA-mediated protection of FtsZ polymers from the negative action of MinC has been reported both for the *B. subtilis* [16] and *E. coli* proteins [17]. In addition to MinC, ZapA also counteracts the inhibition of FtsZ assembly by the SOS response factor SulA [18]. Interestingly, ZapA enhances the effect of Noc, the mediator of nucleoid occlusion in *B. subtilis*, binding to and cross-linking the filaments corralled to midcell by the latter, and actually compensates its absence [19]. There is no information, however, on the interplay between ZapA and the negative regulation of FtsZ polymerization by the nucleoid occlusion system in Gram-negative organisms.

Nucleoid occlusion is a mechanism that inhibits FtsZ from forming a ring near the chromosome to protect the genomic material from physical damage caused by an active division septum [20]. In most Gram-negative bacteria, including *E. coli*, the factor that mediates this mechanism is the DNA-binding protein SlmA [21]. Upon specific interaction with an array of SlmA-binding sequences (SBSs), localized within certain areas of the chromosome outside the terminus region [22,23], SlmA inhibits FtsZ polymerization [22,24] reducing the lifetime of its GTP-triggered polymers [25]. In dilute solution, an SBS oligonucleotide containing a single site binds 4 SlmA monomers, arranged as a couple of dimers that do not interact with each other [25,26]. The formation of this protein/DNA complex is key for the interaction of SlmA with FtsZ and, hence, for the antagonistic action of the nucleoid occlusion factor over the GTP-triggered FtsZ polymerization [22,25].

The nucleoprotein complexes of SlmA also interact with the GDP form of FtsZ [23] and these multivalent interactions, arising from the ability of FtsZ to form oligomers [6] and of the SBS sites to bind various SlmA monomers [26], lead to the formation of phase-separated biomolecular condensates under conditions mimicking the crowded bacterial cytoplasm [7]. Biomolecular condensation through phase separation has emerged as a new principle for the regulation of the intracellular function, originally demonstrated in eukaryotic cells [27] but recently also in bacteria [28]. Condensates accumulate scaffold elements indispensable for their formation, usually proteins with multiple domains of interaction and/or intrinsically disordered regions and sometimes nucleic acids [29]. They also recruit other non-scaffold molecules [30]. The role of biomolecular condensates in bacteria is still elusive but, besides a general role as subcellular organizers of biological function, there is increasing evidence of linkages with bacterial fitness and tolerance to different kinds of stresses [31].

FtsZ-SlmA-SBS condensates are dynamic, reversibly interconvert into filaments in the presence of GTP, and are found preferentially at the membrane when reconstituted into microfluidics cell-like systems [7]. Condensation increases with crowder concentration, and the condensate size decreases with increasing ionic strength, probably related to the electrostatic interactions involved in their assembly [7]. These condensates have been proposed to act as centres to concentrate the three elements, preventing FtsZ from forming a functional ring under non-division conditions and nearby the nucleoid, at non-central locations, during division. Phase separation of the SlmA nucleoprotein complexes with FtsZ was observed, to a greater or lesser extent, under a wide range of conditions typically used to study the bacterial division proteins in solution and in cell-like systems. By contrast, dynamic reversible biomolecular condensates formed solely by FtsZ were only assembled under more restrictive, but still physiological, conditions that strongly enhanced self-association of the GDP form of FtsZ [8]. Condensation of the central bacterial division protein FtsZ suggests that phase separation might be used as a general mechanism for the overall regulation of cytokinesis in bacteria in which other division proteins, in addition to SlmA, could be involved.

Here we have investigated the impact of ZapA on the crowding-driven biomolecular condensates formed by FtsZ-SlmA-SBS in the absence of GTP, and on the inhibition of GTP-triggered FtsZ polymerization by SlmA-SBS, by using a combination of biophysical, imaging and biochemical reconstitution approaches. Our experiments show that ZapA does not undergo condensation on its own but strongly partitions into the condensates of FtsZ with SlmA-SBS reconstituted in crowded media and in cytomimetic microdroplets obtained by microfluidics. This is consistent with our observation that the two regulators can concurrently form higher order complexes with FtsZ in solution and not compete for FtsZ binding. Modulation of FtsZ polymerization by these regulators was dependent on their relative concentrations, and macromolecular crowding reduced the ZapA concentration necessary to significantly counteract the antagonistic action of SlmA-SBS. This research aims to unravel, by reconstitution in synthetic media, the molecular mechanisms underlying the regulation of FtsZ function through specific factors, shedding light on their interplay with two kinds of FtsZ supramolecular structures, biomolecular condensates and polymers.

## 2. Results

### 2.1. ZapA incorporates into homo- and heterotypic FtsZ biomolecular condensates

**Figure 1.**
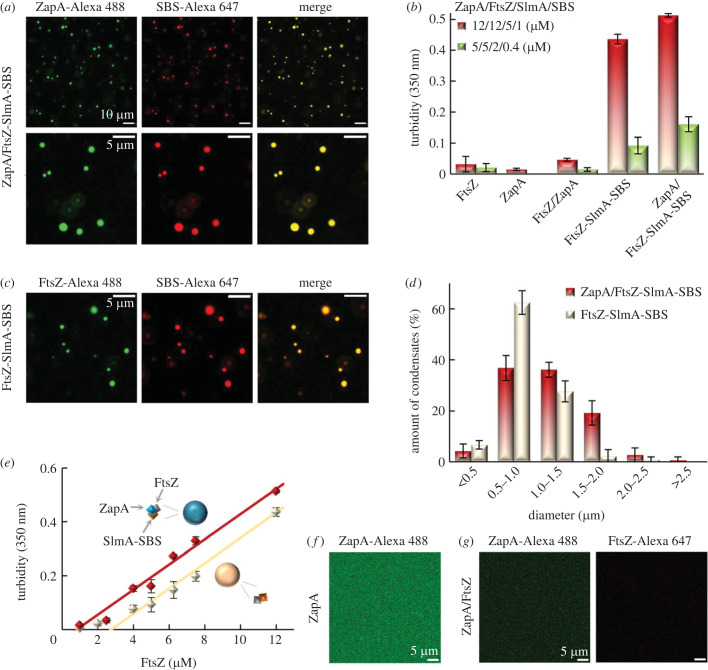
ZapA incorporates into the FtsZ-SlmA-SBS condensates with a subtle enhancing effect. (*a*) Representative confocal images showing accumulation of ZapA in FtsZ-SlmA-SBS condensates. (*b*) Turbidity of samples at two different concentrations. For 12/12/5/1 µM ZapA/FtsZ/SlmA/SBS, *n* = 4 for all samples except FtsZ-SlmA-SBS (*n* = 6) and ZapA/FtsZ-SlmA-SBS (*n* = 3); for 5/5/2/0.4 µM ZapA/FtsZ/SlmA/SBS, *n* = 15 (ZapA/FtsZ-SlmA-SBS), *n* = 9 (FtsZ-SlmA-SBS), *n* = 3 (FtsZ/ZapA) and *n* = 4 (FtsZ). (*c*) Confocal images showing FtsZ-SlmA-SBS condensates. (*d*) ZapA slightly increases the size of condensates. Size distribution of condensates obtained from confocal images in the absence (*n* = 178 particles) and presence of ZapA (*n* = 304 particles). Errors are s.d. from 4 and 6 (without and with ZapA, respectively) independent images. The average number of condensates per image was approximately 50, both in the absence and in the presence of ZapA. (*e*) ZapA slightly decreases the *c*_sat_ of condensation. Dependence of the turbidity signal of FtsZ-SlmA-SBS with and without ZapA with FtsZ concentration. Concentrations were varied keeping a 12 : 5 : 1 (FtsZ : SlmA : SBS) molar ratio, and 1 : 1 ZapA : FtsZ. Data are the average of at least three independent experiments ± s.d. Lines correspond to a linear model fit to the data, to obtain *c*_sat_ values. (*f*,*g*) Absence of condensates in ZapA samples without and with FtsZ, respectively. In all experiments, concentrations were 12 µM (FtsZ and ZapA), 5 µM (SlmA) and 1 µM (SBS) except when stated. When present, labelled components were at 1 µM. All experiments were performed in crowding buffer with 300 mM KCl.

The effect of ZapA on the FtsZ-SlmA-SBS condensates, formed under crowding conditions as previously described [7], was analysed by confocal microscopy and turbidity. Confocal images of samples containing the three proteins and the SBS, in dextran 500 as crowding agent, showed condensates in which ZapA labelled with Alexa 488 (ZapA-Alexa 488) and the SBS labelled with Alexa 647 (SBS-Alexa 647) colocalize (figure 1*a*). Turbidity measurements of these condensates evidenced an absorbance signal slightly higher than that of the condensates without ZapA (figure 1*b*), indicating that this protein may exert a mild enhancing effect on the sizes and/or abundance of the condensates. In support of this idea, the sizes of condensates measured from confocal images (figure 1*a*,*c*) are slightly larger in the presence of ZapA (figure 1*d*), with no obvious difference in the number of condensates. Notably, condensates in which ZapA accumulates were also found when the concentrations of the elements forming them were lowered twofold (electronic supplementary material, figure S1a). As observed at higher concentrations, the turbidity signal slightly increased in the presence of ZapA (figure 1*b*) and, in this case, confocal images showed a modest increase in the number and size of the condensates compared with those formed in the absence of ZapA under the same conditions (*cf*. electronic supplementary material, figures S1a,b). As expected, condensates were also found when the three proteins and the SBS were mixed but none of the elements was fluorescently labelled, in good agreement with turbidity measurements (electronic supplementary material, figure S1c).

Biomolecular condensation phenomena are typically characterized by a saturation concentration (*c*_sat_), a concentration threshold above which phase separation leading to condensates occurs [32,33]. Because determination of this parameter is not straightforward for heterologous condensates [34] such as those formed by FtsZ-SlmA-SBS, we estimated an apparent *c*_sat_ to assess the impact of ZapA (figure 1*e*). Turbidity measurements conducted at increasing concentrations of FtsZ, SlmA and the SBS oligonucleotide while keeping their molar ratio (12 : 5 : 1) constant rendered an apparent *c*_sat_ = 2.8 ± 0.3 µM, in terms of FtsZ concentration, for the condensates in the absence of ZapA. The presence of ZapA at equimolar concentrations with FtsZ resulted in a minor shift of this parameter to lower values (*c*_sat_ = 0.8 ± 0.1 µM). This mild stimulatory effect of ZapA on condensate formation suggests that the protein integrates into the condensates but does not function as a scaffold.

ZapA was also found to strongly partition into condensates formed upon mixing the three proteins and the SBS sequence at higher and lower (500 and 100 mM) salt concentrations (electronic supplementary material, figure S2). Confocal images showed a progressive decrease of the apparent size of the condensates with increasing salt concentration (electronic supplementary material, figure S2a), with a concomitant large decrease in the turbidity signal (electronic supplementary material, figure S2b). This decrease was also observed in the absence of ZapA (electronic supplementary material, figure S2b), in agreement with previously described data [7]. The same trend was observed upon addition of ZapA following, rather than prior to, FtsZ-SlmA-SBS condensation (electronic supplementary material, figure S3a) and at lower protein and DNA concentrations (electronic supplementary material, figure S3b).

ZapA did not form condensates on its own and neither was it able to promote FtsZ condensation under the experimental conditions used to characterize the ZapA/FtsZ-SlmA-SBS condensates, as shown by confocal imaging (figure 1*f,g*; electronic supplementary material, figure S4a,b), in accordance with the almost negligible turbidity signals for ZapA and/or FtsZ (figure 1*b*). By contrast, the addition of SlmA-SBS into samples containing both proteins did trigger the formation of condensates, consistent with the crucial role of the nucleoprotein inhibitor in condensation (electronic supplementary material, figure S4c).

Increasing both the Mg^2+^ and crowding concentrations under low-salt conditions promoted FtsZ homotypic condensation as described [8], while a single continuous phase was still observed for solutions containing only ZapA (electronic supplementary material, figure S5a). As for the FtsZ-SlmA-SBS condensates, confocal images showed that those formed solely by FtsZ, known to coexist under these conditions with a certain amount of irregular assemblies, also incorporated ZapA, whether ZapA was added over preformed condensates of FtsZ or mixed with FtsZ before condensation (electronic supplementary material, figure S5b,c). The addition of ZapA resulted in an increase in the turbidity measured (electronic supplementary material, figure S5d), consistent with an increase in the size and abundance of the FtsZ structures in the images. This suggests ZapA may have a more important influence in FtsZ condensation than that observed when SlmA-SBS is also present. Addition of SlmA-SBS into the FtsZ condensates promoted both an increase in their apparent sizes and abundance (electronic supplementary material, figure S5e).

These experiments show that ZapA accumulates in phase- separated biomolecular condensates formed by FtsZ-SlmA-SBS under a broad range of conditions and in condensates formed by FtsZ under more limited conditions. The mild enhancing effect of ZapA on the tendency of FtsZ-SlmA-SBS to phase-separate, together with its inability to form condensates on its own or to induce FtsZ condensation, suggest that incorporation of ZapA into the condensates, most likely through direct interaction with FtsZ, does not involve its functioning as a scaffold.

### 2.2. ZapA/FtsZ-SlmA-SBS condensates are dynamic and reversible

**Figure 2.**
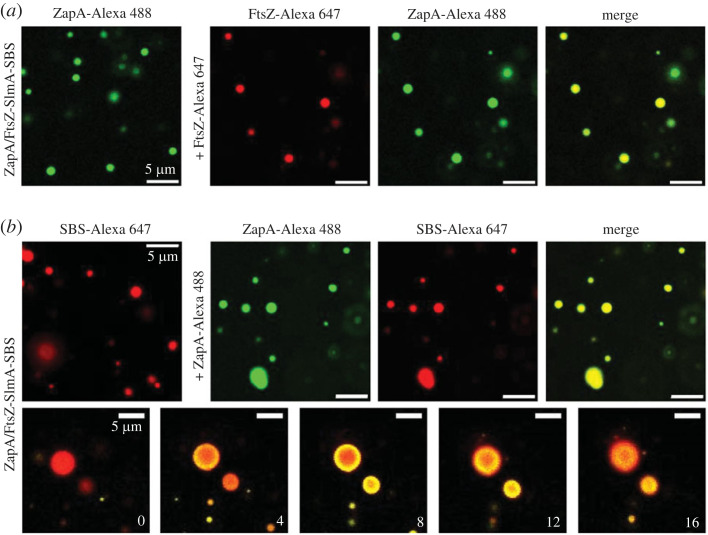
ZapA/FtsZ-SlmA-SBS condensates are dynamic. (*a*) Representative confocal images showing capture of freshly added FtsZ-Alexa 647 by ZapA/FtsZ-SlmA-SBS condensates labelled with ZapA-Alexa 488. (*b*) Images showing incorporation of ZapA-Alexa 488 to ZapA/FtsZ-SlmA-SBS condensates labelled with SBS-Alexa 647. Below, images showing the stepwise diffusion of ZapA-Alexa 488 into the condensates at the indicated times in seconds (time zero, beginning of visualization for those particular condensates). Concentrations were 12 µM (FtsZ and ZapA), 5 µM (SlmA) and 1 µM (SBS and labelled elements). Experiments were conducted in crowding buffer with (*a*) 300 mM KCl or (*b*) 100 mM KCl.

Dynamism of condensates is a feature widely accepted to define these structures [27,29]. In order to ascertain whether the FtsZ-SlmA-SBS condensates remain dynamic [7] despite the integration of ZapA, we conducted capture experiments with either ZapA or FtsZ. Addition of FtsZ labelled with Alexa 647 (FtsZ-Alexa 647) into ZapA/FtsZ-SlmA-SBS condensates labelled with ZapA-Alexa 488 resulted in the colocalization of both signals in confocal images of the final state, indicating the freshly added protein incorporated into the already formed condensates (figure 2*a*). Likewise, ZapA-Alexa 488 dynamically and progressively incorporated into the ZapA/FtsZ-SlmA-SBS condensates labelled with SBS-Alexa 647 (figure 2*b*), further underscoring the dynamism of the nucleoprotein condensates incorporating ZapA. In addition to being dynamic, condensates were also observed to reversibly assemble in response to changes in salt content. Rapid dissociation of condensates formed at 300 mM KCl and shifted to 500 mM substantially reduced the associated turbidity signal, matching values of condensates directly formed at 500 mM KCl (electronic supplementary material, figure S2b).

### 2.3. Non-competing binding of ZapA and SlmA to FtsZ oligomers

To better understand the effect of ZapA on the FtsZ-SlmA-SBS condensates, we evaluated the interactions involving these three proteins and the SBS sequence by using orthogonal analytical ultracentrifugation (sedimentation velocity, SV, and equilibrium, SE) and fluorescence-based (fluorescence correlation spectroscopy, FCS, and anisotropy) methods. As a prior step, we analysed the oligomerization state of ZapA under our experimental conditions. ZapA underwent the expected dimer-tetramer self-association, which we found was regulated by salt (see electronic supplementary material, figure S6 and related text). This suggests an important contribution of electrostatic forces, in good agreement with the charged residues in the helices of the ZapA dimers that interact to form the tetramer [35].

**Figure 3.**
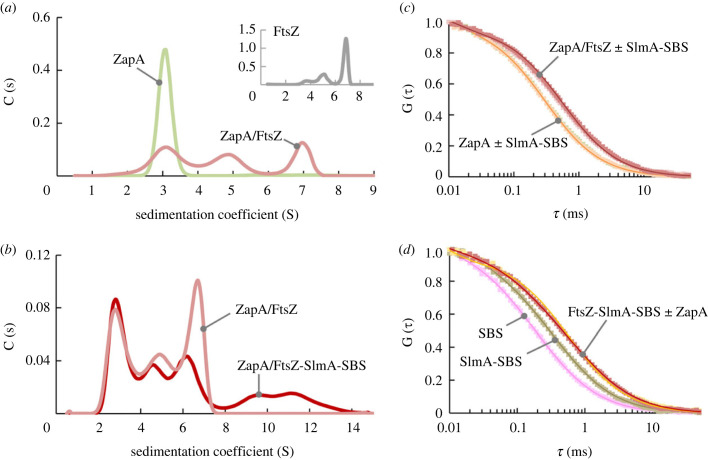
Non-competing interaction of ZapA and SlmA-SBS with FtsZ oligomers. (*a*) ZapA interacts with FtsZ oligomers of different sizes. Distribution of ZapA species (5 µM, with 2.7 µM ZapA-Alexa 488) without and with FtsZ (60 µM) obtained from SV followed at 488 nm. Inset shows FtsZ distribution, in the absence of ZapA, followed at 280 nm, for comparison. (*b*) Formation of higher order complexes upon addition of SlmA-SBS to ZapA/FtsZ. SV analysis of ZapA-Alexa 488 (2.6 µM) in samples of ZapA/FtsZ (5 and 40 µM, respectively) without and with SlmA-SBS (10 and 2 µM, respectively), followed at approximately 495 nm. (*c*,*d*) Absence of competition between ZapA and SlmA-SBS for FtsZ oligomers in FCS experiments. (*c*) From faster to slower diffusion, normalized FCS autocorrelation curves of ZapA without and with SlmA-SBS, and ZapA/FtsZ in the absence and presence of SlmA-SBS. Curves of ZapA with FtsZ ± SlmA-SBS are indistinguishable, as well as those corresponding to ZapA ± SlmA-SBS. Solid lines are the fits of the model indicated in the electronic supplementary material. All samples contained 10 nM ZapA-Alexa 488. (*d*) From faster to slower diffusion, normalized FCS autocorrelation curves of SBS without and with SlmA, and FtsZ-SlmA-SBS without and with ZapA. Profiles of SlmA-SBS with FtsZ ± ZapA overlapped. All samples contained 10 nM SBS-Alexa 488. Concentrations in (*c*) and (*d*) were 5 µM ZapA, 5 µM SlmA, 50 µM (*c*) or 20 µM (*d*) FtsZ, and 1 µM SBS. All experiments were done in solution buffer with 100 mM KCl.

Both ZapA and SlmA-SBS complexes have been previously shown to recognize FtsZ in the absence of GTP [18,23,25]. The question arises whether both ZapA and the SlmA-SBS complex, as positive and negative regulators, respectively, compete or can bind simultaneously to FtsZ. To address this, we started by setting up an assay based on SV to monitor the complexes involving ZapA and FtsZ. SV analysis of FtsZ showed the formation of oligomers of different sizes (inset in figure 3*a*), as previously described [6]. Profiles obtained from samples containing ZapA-Alexa 488 (to specifically monitor the species containing this protein) and FtsZ displayed various peaks besides that corresponding to free ZapA (approx. 3 S; figure 3*a*), at *s*-values similar to those found for FtsZ alone. This indicated that ZapA binds to the different FtsZ oligomers (for more details, see electronic supplementary material, figure S7 and related text). We then repeated the SV experiments, incorporating SlmA-SBS in the mixtures of FtsZ and ZapA (with ZapA-Alexa 488). In the profiles (figure 3*b*), the peak at approximately 3 S corresponding to free ZapA remained virtually unaltered in the presence of SlmA-SBS, suggesting that the nucleoprotein complex does not interact with ZapA and, more importantly, that it does not compete with ZapA for binding FtsZ within the complexes. In addition to the peaks of free ZapA and the FtsZ-ZapA complexes, broad peaks at *s*-values above 8 S were observed in this profile, not found in the absence of the nucleoprotein complexes, probably corresponding to species containing the three proteins (figure 3*b*).

FCS experiments also supported the absence of competition between the regulators under the assayed conditions. Addition of FtsZ to ZapA (with ZapA-Alexa 488 as a tracer) resulted in a shift of the FCS autocorrelation curves to longer timescales due to the formation of heterocomplexes (electronic supplementary material, figure S7b). The apparent translational diffusion coefficients ranged from approximately 60 µm^2^ s^−1^ in the absence of FtsZ, compatible with ZapA tetramers (see electronic supplementary material, figure S6 and related text), to approximately 35 µm^2^ s^−1^ at the highest concentration of FtsZ tested (electronic supplementary material, figure S7c). Under the assayed conditions, profiles corresponding to the samples containing ZapA-Alexa 488 and FtsZ were insensitive to the addition of SlmA-SBS (figure 3*c*), strongly suggesting that the amount of free ZapA did not increase in the presence of SlmA-SBS. According to our FCS experiments, ZapA did not displace SlmA from the complexes with FtsZ either. Autocorrelation curves determined for the FtsZ-SlmA-SBS complexes using SBS-Alexa 488 showed the formation of complexes, resulting in a shift of the apparent diffusion coefficient for the SlmA-SBS complexes from 50.5 ± 0.5 µm^2^ s^−1^ in the absence of FtsZ to 31 ± 1 µm^2^ s^−1^ in its presence (figure 3*d*). Addition of ZapA did not modify the profiles, which is again consistent with non-competitive behaviour. FCS experiments were also compatible with a lack of interaction between ZapA and SlmA-SBS in the absence of FtsZ, as expected (figure 3*c*).

Therefore, both ZapA and SlmA are able to bind FtsZ oligomers and, under the conditions used in these assays, they did not exhibit competitive behaviour, forming complexes presumably involving the three proteins and the nucleic acid sequence.

### 2.4. ZapA/FtsZ-SlmA-SBS condensates interconvert with GTP-induced polymers in which the two regulators are simultaneously present

A feature defining biomolecular condensates is their reversible formation depending on the environmental conditions, and their ability to respond to specific physiological ligands. In the case of FtsZ condensates, previous studies have shown that FtsZ leaves the FtsZ-SlmA-SBS condensates in the presence of GTP, which triggers its polymerization. FtsZ assembles back into condensates when bundles of polymers, formed by lateral association of the polymers under crowding conditions, disband due to GTP hydrolysis [7]. We thus proceeded to verify whether this behaviour was maintained in the condensates that also incorporated ZapA.

**Figure 4.**
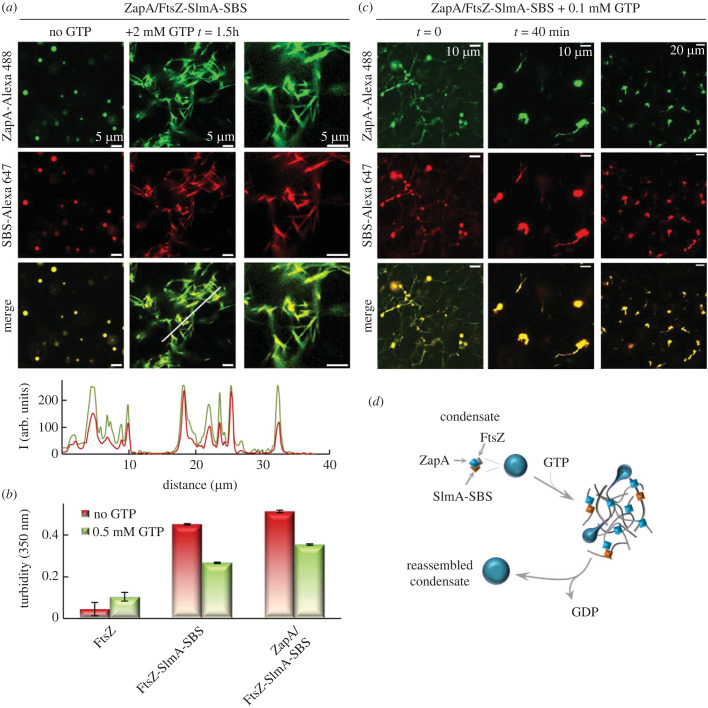
FtsZ within ZapA/FtsZ-SlmA-SBS condensates forms polymers in response to GTP addition. (*a*) Representative confocal images of ZapA/FtsZ-SlmA-SBS condensates showing the formation of polymers after addition of GTP. Below, intensity profiles corresponding to the green and red channels were obtained along the line drawn in the associated image. (*b*) Decrease in turbidity indicative of polymers coming from condensates. Depicted are the turbidity values of samples of FtsZ (*n* = 2) and condensates with (*n* = 3) and without ZapA (*n* = 2) before (samples incubated for 30 min) and after addition of GTP (values taken immediately after addition of nucleotide on the incubated samples). (*c*) Images of ZapA/FtsZ-SlmA-SBS at time zero and 40 min after the addition of GTP. In (*a*) and (*c*), column in the far right corresponds to a different magnification of the middle column. (*d*) Scheme depicting the reversible interconversion between condensates and bundles of FtsZ with the modulators. In all experiments, concentrations were 12 µM (FtsZ and ZapA), 5 µM (SlmA) and 1 µM (SBS and labelled elements). Experiments were carried out in crowding buffer with 300 mM KCl.

Addition of GTP to ZapA/FtsZ-SlmA-SBS condensates readily triggered assembly of FtsZ polymers at the expense of the condensates (figure 4*a*), confirming FtsZ remains active in these condensates as well. Confocal images showed colocalization of ZapA-Alexa 488 with SBS-Alexa 647 in the polymers (figure 4*a*). This is compatible with the two partners simultaneously binding FtsZ polymers (although not necessarily through interaction with the same monomer), as previously found in the condensates (see above). We note that the extent of colocalization depended on the time elapsed after GTP addition. Polymerization of FtsZ-SlmA-SBS condensates was accompanied by a decrease in the turbidity signal both in the absence and presence of ZapA (figure 4*b*). The turbidity of FtsZ samples that, under these working conditions, are not forming condensates, varied in the opposite sense (figure 4*b*) as expected for the emergence of large bundles from discrete oligomeric species. Decrease in the turbidity values supports disassembly of the condensates concomitantly with FtsZ polymerization because, otherwise, the turbidity arising from the polymers would have added up to that of the condensates, increasing the net signal. Lower concentrations of GTP allowed visualizing the polymers entangled with, and presumably protruding from, the condensates (figure 4*c*).

In the absence of the regulators, FtsZ formed a homogeneous network of bundles whose appearance and distribution did not seem to be substantially altered by ZapA, which colocalized with FtsZ all along them (electronic supplementary material, figure S8a,b). As previously described and in line with its antagonistic effect, the FtsZ bundles were less abundant, distributed more heterogeneously and appeared thinner and less defined when only SlmA-SBS was present, added either before or shortly after FtsZ polymerization (electronic supplementary material, figure S8b,c). This local accumulation might be related to its destabilization mechanism that yields shorter polymers [25], rather than producing their complete dissociation, and with the fact that the FtsZ bundles containing SlmA are in equilibrium with condensates. In the presence of ZapA, the negative effect of SlmA-SBS appeared to be somewhat counteracted, the polymers being better defined in the images (figure 4*a*; electronic supplementary material, figure S8d). With SlmA-SBS, the homogeneous distribution of ZapA was not affected (cf. electronic supplementary material, figure S8a,e).

FtsZ polymers induced by GTP (0.5 mM) added to FtsZ-SlmA-SBS condensates were previously shown to disassemble because of the action of the SlmA-SBS inhibitor, fully reassembling back into condensates in less than 10 min [7]. In the presence of ZapA, confocal images showed that the condensates had still not fully reassembled 40 min after polymerization, under the same conditions as in the former study but with only 0.1 mM GTP (figure 4*c*). This suggests that reassembly of condensates is delayed by the presence of ZapA (i.e. ZapA stabilizes the polymers), as confirmed by fluorescence anisotropy measurements (see below). A reasonable explanation for this stabilization would be the cross-linking effect of ZapA, which would likely counteract the antagonistic action of SlmA on the polymers, as also suggested above from the confocal images (figure 4*a*; electronic supplementary material, figures S8d,e versus S8b,c).

Immediate formation of polymers and full reassembly of condensates from polymers after GTP exhaustion was also observed at lower protein (electronic supplementary material, figure S9) or salt concentrations (electronic supplementary material, figure S10). In 100 mM KCl, condensates remained numerous together with the polymers at the time imaged, and reassembled structures after GTP depletion were substantially larger (electronic supplementary material, figure S10).

These experiments show that FtsZ-SlmA-SBS condensates interconvert with polymers depending on the GTP/GDP ratio (i.e. in response to GTP addition and depletion) also in the presence of ZapA, and that this protein can accumulate in both structures. The division proteins within the condensates retain their functional hallmarks, namely the ability of FtsZ to reversibly polymerize, the inhibition of FtsZ polymerization by SlmA-SBS complexes that ultimately promotes rapid condensate reassembly, and the stabilization of FtsZ polymers by ZapA, opposing their dissociation and subsequent condensate formation.

### 2.5. Crowding largely reduces the ZapA : SlmA-SBS ratio dictating ZapA-mediated stabilization of FtsZ polymers

To further dissect the antagonistic roles of the two FtsZ regulators, we analysed their interactions with the GTP-triggered FtsZ polymers by following a combined biophysical approach similar to that described in the presence of GDP (see above). Although information on the interaction of ZapA with FtsZ polymers is already available, data under our specific conditions were gathered (see electronic supplementary material, figure S11 and related text) as it would benefit the characterization of the interplay of this pair with SlmA-SBS, given the sensitivity of FtsZ and its complexes to the buffer composition. Results obtained by FCS and SV are consistent with the formation of complexes of ZapA with the GTP-induced FtsZ polymers, resulting in an increase in the average mass of the polymers as a function of ZapA concentration (electronic supplementary material, figure S11 and related text). SlmA-SBS binding to, and modulation of, FtsZ polymers has been previously analysed under conditions similar to those used here [25].

**Figure 5.**
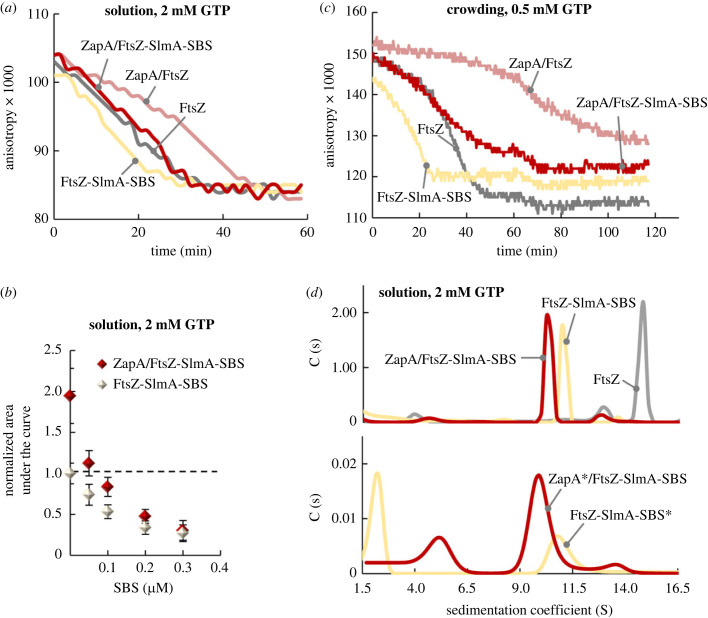
Concentration-dependent control of FtsZ polymerization by ZapA and SlmA-SBS. (*a*) ZapA and SlmA stabilize and destabilize FtsZ polymers, respectively. Representative depolymerization profiles of FtsZ in the presence of the SlmA-SBS complex, ZapA or both, monitored by fluorescence anisotropy right after addition of GTP. (*b*) Calculated area under the anisotropy profiles in (*a*) and in electronic supplementary material, figure S12a as a function of SlmA-SBS concentration, in the absence and presence of ZapA. Values are the average of three independent measurements ± s.d. Areas are normalized regarding FtsZ values in the absence of any regulator (depicted by a horizontal dashed line). (*c*) Crowding enhances the effect of ZapA against SlmA-SBS acceleration of FtsZ disassembly. Representative depolymerization profiles in the presence of 150 gl^−1^ dextran of FtsZ with SlmA-SBS complex, ZapA or both, monitored by fluorescence anisotropy right after addition of GTP. Concentrations in (*a*)–(*c*) were 5 µM FtsZ (50 nM FtsZ-Alexa 488), 5 µM ZapA and 0.05 µM (*a*), 0.3 µM (*c*) or increasing concentrations (*b*) of the SlmA-SBS complex. (*d*) SlmA-SBS and ZapA bind to FtsZ polymers. SV interference distributions of FtsZ and SlmA-SBS with and without ZapA, in the presence of GTP and RS. The profile obtained for FtsZ in the absence of the modulators is also shown for reference. Below, SV distributions obtained for these samples following the absorbance at 490 nm of 1.45 µM ZapA-Alexa 488 or 1.2 µM SBS-Fl (fluorescent dyes depicted with an asterisk). Concentrations were 10–12 µM FtsZ, 6.25 µM SlmA, 1.2 µM SBS and 3 µM ZapA. All experiments were done in solution buffer with 300 mM KCl.

Evaluation of the effects of both regulators on FtsZ assembly was conducted by fluorescence anisotropy with FtsZ-Alexa 488 as a tracer, which allows detection of filament formation and monitoring of their time-dependent disassembly. In the absence of the regulators, addition of GTP to FtsZ resulted in a jump in anisotropy due to the formation of polymers, which subsequently decreased to the basal value within approximately 35 min due to GTP hydrolysis (figure 5*a*). In each other's absence, SlmA-SBS and ZapA reduced and prolonged, respectively, the time period in which FtsZ polymers were detected (figure 5*a*). The concentration-dependent shortening of the duration of the polymers by SlmA-SBS was previously reported [25]. In the case of ZapA, the longer duration of the polymers would be related with the known reduction of FtsZ GTPase activity by this regulator [36], the extent of the effect being also related to the amount of ZapA (electronic supplementary material, figure S11d,e).

Samples with constant equimolar concentrations of FtsZ and ZapA (5 µM) and variable concentration of the SlmA-SBS complex were also measured (figure 5*b*; electronic supplementary material, figure S12a). At low concentrations of the nucleoprotein complex, the induced shortening of polymer lifetimes was significantly counteracted by ZapA. At 100-fold excess of ZapA (0.05 µM SlmA-SBS), the effect of the regulators virtually cancelled each other out, so the value obtained matched that of FtsZ polymers alone (i.e. the magnitudes of the stabilization by ZapA and the destabilization by SlmA-SBS were similar). The polymers were only minimally stabilized against the action of SlmA-SBS with a 25-fold excess of ZapA (0.2 µM SlmA-SBS), and with an approximately 17-fold excess (0.3 µM SlmA-SBS) polymers were insensitive to the addition of ZapA, highlighting the strong effect of SlmA-SBS as an antagonist of FtsZ polymerization.

Interestingly, the ZapA : SlmA-SBS ratios required for significant counteraction of the antagonistic effects of the latter in dilute solution largely exceeded those assayed in condensate/bundle interconversion analysis under crowding conditions (typically 12 : 1), where the ZapA protection was already detected (see above). To shed light on this apparent discrepancy, anisotropy experiments under crowding conditions were performed, at protein ratios matching those of the confocal microscopy images (electronic supplementary material, figure S12b), evidencing a clear stabilization of the polymers by ZapA at these lower ratios, in agreement with the imaging experiments (figure 4; electronic supplementary material, figure S8). This strongly suggested that macromolecular crowding conditions could reinforce the agonistic action of ZapA. To further prove this point, the above-described anisotropy experiments at 17 : 1 ZapA : SlmA-SBS (0.3 µM SlmA-SBS) were repeated in the presence of dextran (figure 5*c*). In contrast with the insensitivity to ZapA in diluted solution at these concentrations, significant stabilization was found under crowding conditions, confirming the synergy between crowding and ZapA to enhance the protection of FtsZ polymers.

Mutual counteraction of their effects could be explained by competition between both regulators for binding to FtsZ, or with any other mechanism involving their simultaneous binding to the same or different subunits within the FtsZ polymers that hindered each other's action. Our results support the second mechanism, as we could not detect competition. Initial evidence was obtained from SV experiments of samples containing FtsZ, ZapA and SlmA-SBS plus GTP (figure 5*d*). At the low-ZapA concentration relative to that of SlmA-SBS, the sedimentation coefficient distribution obtained by interference (figure 5*d*, top) was equivalent to that observed in the absence of ZapA (i.e. the effect of SlmA-SBS prevailed). Thus, there was a considerable shift of the peak corresponding to the FtsZ polymers from approximately 15 S to approximately 10–11 S. Interestingly, despite the insensitivity to the presence of ZapA, when following the absorbance signal of an Alexa 488 dye attached to this protein (figure 5*d*, bottom), it also peaked at approximately 10 S, evidencing its presence in the smaller species resulting from the inhibitory action of SlmA-SBS. A parallel experiment using SBS labelled with fluorescein (SBS-Fl) confirmed that the nucleoprotein complex remained bound to these species as well (figure 5*d*, bottom). We note that higher order complexes were formed in these experiments, as we detected a signal reduction when reaching working speed of the SV experiment.

This analysis revealed that the antagonistic effects of SlmA-SBS and ZapA on FtsZ polymerization rely on their relative concentrations, and that high excess of ZapA is required to hinder inhibition by the nucleoprotein complexes in dilute solution. The mechanisms underlying the interplay between these regulators do not seem to be based on competition for binding to FtsZ. Crowding conditions strengthen the action of ZapA, diminishing the levels of this protein required to significantly revert the negative regulation of FtsZ assembly by SlmA-SBS.

### 2.6. Reconstitution of FtsZ with the two regulators in cell-like systems

Previous work demonstrated that FtsZ-SlmA-SBS could form condensates in cell-like systems stabilized by a lipid layer that mimicked the composition of the *E. coli* inner membrane and featured crowding and compartmentalization in the lumen [7]. Following an analogous microfluidic encapsulation procedure, we generated microdroplets with dextran as a single crowder to ascertain the impact of ZapA on the behaviour of the condensates.

**Figure 6.**
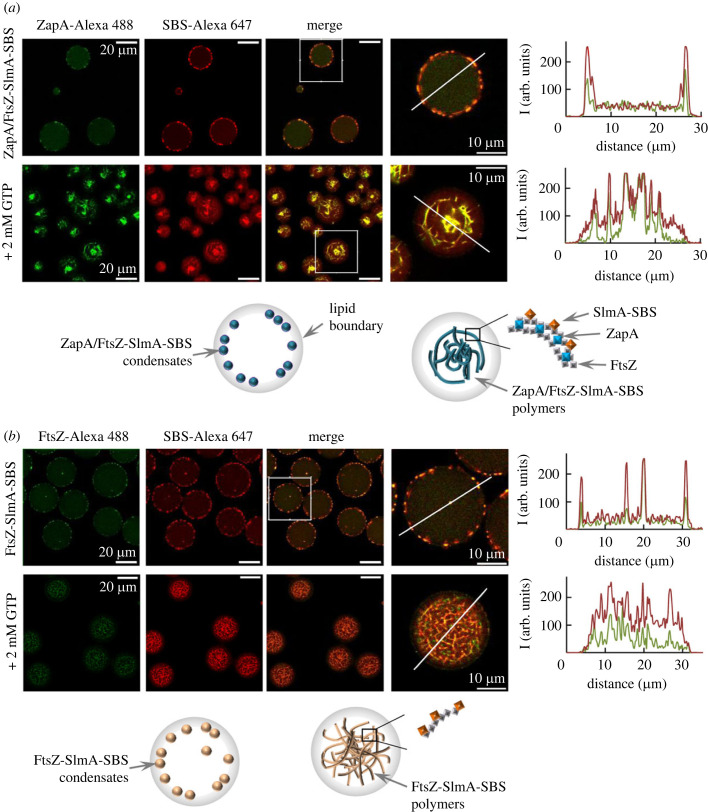
Reconstitution of ZapA/FtsZ-SlmA-SBS condensates and polymers in lipid-stabilized microfluidics microdroplets. Representative confocal images of microdroplets containing FtsZ-SlmA-SBS condensates with (*a*) and without ZapA (*b*), showing the formation of condensates (top) or GTP-triggered polymers (bottom), and schematics illustrating their distribution below. On the far right, magnification of the indicated section in the merged image, except for bottom row in (*b*) showing an independent image with higher magnification. Intensity profiles corresponding to the green and red channels, obtained along the line drawn in the images, are also shown. Concentrations were 12 µM (FtsZ and ZapA), 5 µM (SlmA) and 1 µM (SBS and labelled elements). All experiments were performed in crowding buffer with 300 mM KCl.

Encapsulation of FtsZ, SlmA, SBS-Alexa 647 and ZapA (with ZapA-Alexa 488 as tracer) resulted in the formation of ZapA/FtsZ-SlmA-SBS condensates in which ZapA and SBS colocalize, similarly to that found in crowding conditions in bulk. Condensates were mostly located at the lipid interface of the microdroplets (top row in figure 6*a*), with some non-specific localization to the lumen. Experiments conducted using labelled FtsZ and ZapA rendered similar results (electronic supplementary material, figure S13a). Without ZapA, condensates showed the same preferential location at the lipid surface (top row in figure 6*b*), in line with previous results in microdroplets with two compartments obtained by co-encapsulation with crowders exhibiting phase separation [7]. As in bulk solution, encapsulated ZapA did not form condensates on its own (electronic supplementary material, figure S13b), and it was not able, either, to induce FtsZ condensates (electronic supplementary material, figure S13c).

The FtsZ polymers were also reconstituted in lipid-stabilized microdroplets containing dextran, inducing polymerization with GTP just before encapsulation, to analyse the impact of the two regulators on their localization, morphology and stability. Microfluidics encapsulation presented significant advantages for the evaluation of these parameters compared to the above-described analysis of the polymers in bulk solution. Among them, solutions injected in the microfluidic device are thoroughly mixed, and a single experiment yields multiple replicates that are visualized at the same time point after the induction of polymerization, thus leading to more robust conclusions. Using this approach, we found that FtsZ polymers became thicker in the presence of ZapA (bottom rows in electronic supplementary material, figure S13c,d) as expected because of ZapA's cross-linking activity. It is noteworthy that the encapsulated FtsZ polymers in the absence of ZapA were distributed throughout the microdroplet lumen and membrane surface, in agreement with that previously observed [37], whereas FtsZ polymers bundled by ZapA mainly localized in the lumen (electronic supplementary material, figure S13c,d).

In the presence of SlmA-SBS, the addition of GTP preceding microdroplet formation triggered the assembly of FtsZ polymers, with or without ZapA (bottom rows in figure 6*a*,*b*). FtsZ polymers formed under the control of the nucleoprotein antagonist were uniformly distributed as an intricate network throughout the entire microdroplet volume (figure 6*b*). In the presence of the positive regulator, the polymers were located mainly in the lumen, apparently expelled from the membrane and arranged into larger structures (figure 6*a*). As observed in bulk solution, ZapA and the SBS also colocalized in the encapsulated polymers, which were less homogeneously distributed than when only ZapA or only SlmA-SBS were present (figure 6*a*,*b*; electronic supplementary material, figure S13c).

These experiments show the formation of biomolecular condensates involving FtsZ, the two regulators and the nucleic acid sequence in cell-like systems, highlighting the role of the membrane as an enhancing factor. They also evidence the colocalization between ZapA and SlmA-SBS within FtsZ condensates and polymers in these confined systems, further supporting the formation of structures involving all the elements, rather than competition between both modulators for binding to FtsZ. Analysis of the impact of the regulators on FtsZ polymers distribution and morphology is in line with a protective effect of ZapA against the negative regulation of FtsZ polymers by SlmA-SBS under the assayed conditions.

## 3. Discussion

**Figure 7.**
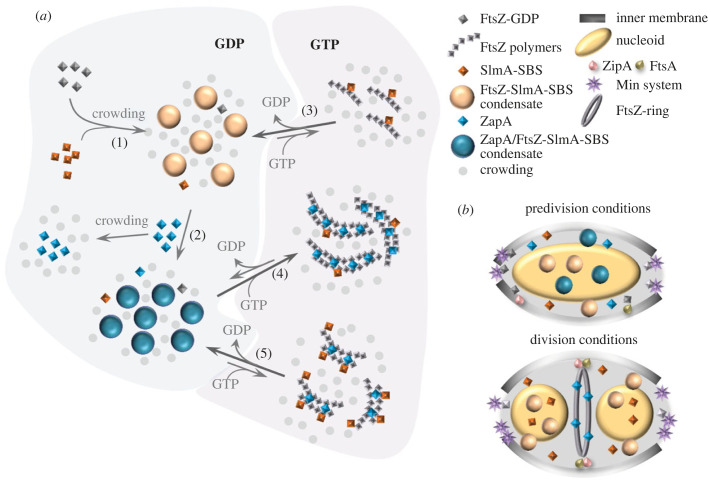
Interplay between bacterial division factors, biomolecular condensates, polymers (and nucleotides) and potential implications in cytokinesis. (*a*) Under cytomimetic crowding conditions, FtsZ forms biomolecular condensates in the presence of SlmA-SBS (1), and ZapA preferentially partitions into them, slightly enhancing condensation (2). GTP triggers FtsZ polymerization from the condensates, which reassemble upon nucleotide depletion. Interconversion between polymers and condensates is modulated by the relative ZapA/SlmA-SBS levels. Thus, SlmA reduces the size of the FtsZ polymers and favours rapid condensate reassembly (3). At high ZapA/SlmA-SBS ratio (4), ZapA significantly counteracts the negative regulation of FtsZ polymerization by SlmA-SBS, delaying condensate reassembly. At lower excess of ZapA relative to SlmA-SBS, polymer destabilization prevails (5). (*b*) In the cell, at stages other than division, the formation of condensates involving FtsZ and SlmA-SBS would strengthen the interactions with the antagonist, efficiently blocking ring formation. Recruitment of ZapA to these assemblies would further prevent ring assembly, as its agonistic action would be neutralized by the strong nucleoprotein antagonist. When cell division is imminent, SlmA will be depleted from the cell centre, due to the orientation of the segregated nucleoids, with their Ter-region devoid of SBS sites facing towards this location. Increase in the concentration of ZapA at the same region would allow achieving the ZapA concentration threshold required to overcome SlmA-SBS inhibition, facilitating FtsZ assembly into polymers cross-linked by ZapA suitable for Z-ring formation, anchored to the membrane by ZipA and FtsA. Outside the division site, the FtsZ-SlmA-SBS condensates may still be present, together with the Min system, to block ring assembly at inappropriate locations in the cell by the significant fraction of FtsZ remaining there.

Here we provide insights into the molecular mechanisms underlying the coordinated regulation of Z-ring formation by two antagonistic modulators, ZapA and SlmA, derived from their reconstitution alongside FtsZ in cytomimetic media. Our analysis suggests that this regulation may be exerted by tuning the equilibria between FtsZ oligomers, biomolecular condensates and the canonical FtsZ polymers known to be at the heart of the Z-ring (figure 7*a*). According to our results, the relative concentrations of the two modulators and weak non-specific interactions arising from the crowded nature of the cytoplasm would largely determine which of the FtsZ species prevail and, hence, the probability of successful Z-ring formation.

We have found that ZapA preferentially partitions into FtsZ-SlmA-SBS condensates, probably as a client protein [30] as it cannot induce condensates or form them and does not significantly influence FtsZ or FtsZ-SlmA-SBS condensates size and *c*_sat_ value. Biophysical analysis of either FtsZ oligomers or polymers with ZapA and SlmA-SBS indicates that they can form ternary complexes in both cases, suggesting that the poorly described binding regions for both regulators in FtsZ do not overlap, although interaction with different subunits within the FtsZ assemblies is also possible. In contrast with ZapA presumably acting as a client, the scaffold role of SlmA-SBS appears beyond doubt given its clear enhancement of FtsZ condensates and the permissiveness for their formation [7]. ZapA incorporation has, however, a clear impact on the dynamics of interchanging FtsZ subunits from filaments to condensates, stabilizing the former against the action of SlmA by presumably cross-linking them [18]. High excess of ZapA is required to counteract the strong inhibition of FtsZ filaments by SlmA-SBS, which could be related to its lower estimated cellular concentration compared to ZapA [21,38]. The substantial reduction under crowding conditions of the ZapA : SlmA-SBS ratio at which the protective effect of ZapA predominates suggests tuning of the polymer-condensate conversion might be determined by swift, subtler concentration changes within the cell, where the local concentrations of ZapA and SlmA-SBS are probably more relevant, with ZapA being much more prevalent at midcell than SlmA-SBS (see below). Examples have been described in which condensate composition changes are related to local fluctuations in concentration (neuronal granules and condensates in fungal cells [39,40]) and function (signalling pathways associated with membrane clusters [30]).

We recently proposed that biomolecular condensates of FtsZ may play a role in the regulation of its interactions with other proteins, localization and cellular distribution and, hence, its function in division [7,8]. Our current results reveal an interplay with the positive regulator ZapA that might endow them with additional levels of modulation. Binding of ZapA and SlmA-SBS to FtsZ oligomers and condensates support the idea that both structures might serve as reservoirs in the cell (figure 7*b*). In quiescent cells and in early parts of the *E. coli* cell cycle during slow growth, FtsZ would likely remain as an unassembled population, as short polymers [41], or would form condensates on its own or (more probably) together with SlmA-SBS. The latter would locate nearby the membrane [7] where they could reinforce the blockage of Z-ring assembly by the Min system. Meanwhile, ZapA would be distributed throughout the cytoplasm [15] and incorporated into FtsZ condensates, including those with SlmA, where ZapA's stimulatory effect on FtsZ polymer assembly would be efficiently counteracted by the highly concentrated inhibitor.

Upon initiation of cell division, a number of events would favour FtsZ release from the condensates with SlmA into polymers suitable for Z-ring formation, stabilized by factors like ZapA. Segregation of the newly replicated chromosome would reduce the concentration of SlmA at midcell, because most of the chromosomal SBS sequences would follow the replication origins and move away from midcell, leaving only the SBS-deficient Ter macrodomain near the middle [22]. ZapA, on the other hand, concentrates between segregating nucleoids even before Z-ring formation [42]. This results in an increase in the ZapA/SlmA-SBS ratio at this location, which would enhance local stable GTP-dependent FtsZ polymerization and concomitant condensate disassembly. An advantage of FtsZ polymers arising from condensates that already contain ZapA versus those in which binding has yet to occur is that their probability of being recruited to the division site through the Ter-linkage is increased. This is because ZapA, directly or indirectly, interacts with its partner proteins including MatP, which binds DNA within the Ter macrodomain. FtsZ filaments cross-linked by ZapA [11,18,38] would attach to the bacterial membrane through ZipA and FtsA [1,2], the natural anchors of the Z-ring in *E. coli*. GTP hydrolysis would free FtsZ subunits that will add up to the significant fraction of FtsZ outside the Z-ring (around two thirds of the total [5,43]), whose misplaced polymerization will be blocked through FtsZ-SlmA-SBS condensation and by the Min system [14]. Further work is required to determine how this behaviour would be modulated not only by the remaining elements of the Ter-linkage, but also by other proteins interacting with FtsZ (e.g. ZipA, FtsA and FtsK) and by those inner membrane divisome proteins that may associate with ZapA but not FtsZ (FtsQ, FtsL, FtsB, FtsW and FtsN) [44–47].

Our reconstitution assays showed that FtsZ-SlmA-SBS can form condensates with ZapA in cytomimetic systems with essential cellular features confined by a lipid membrane, suggesting that these structures may indeed form *in vivo*. Assemblies compatible with biomolecular condensates that, although of unknown composition, contain SlmA in *E. coli* [13,21] and its counterpart in *B. subtilis*, Noc [48,49], and FtsZ in *E. coli* [3,50,51] have been described. In some cases, the putative condensates seem to coexist with FtsZ rings in growing *E. coli* cells [3,52] or in *B. subtilis* cells treated with sub-inhibitory levels of a small molecule compound that binds to FtsZ and reduces its dynamics, potentially favouring the condensate state even in otherwise optimal growth conditions [53]. In other cases, foci that may be condensates appear in cells under non-growing conditions or under stress, disassembling when the stress condition is over and normal growth resumed [50,51]. Moreover, surrogate large cell systems have also suggested that FtsZ can form biomolecular condensates inside cells. When overexpressed in eukaryotic cells, fluorescently tagged *E. coli* FtsZ assembled into dozens of distributed foci throughout the cytoplasm that localized independently of the mammalian cytoskeleton [54]. Upon exposure to the anti-tubulin drug vinblastine, FtsZ polymers emerged from the foci. After disassembly of the FtsZ polymers, these foci reappeared [54], indicating that FtsZ can interconvert between a condensate-like storage form and dynamic polymers. Because the term ‘biomolecular condensate’ was only relatively recently coined, bacterial structures identified in earlier works as foci, puncta, dots, regrowth delay bodies, etc. were not considered as such. This, together with the optical limitations in visualizing condensates in these small cells might have caused the largely overlooked description of these structures in bacteria.

In addition to their specific functional roles, a growing body of research supports the role of bacterial condensates in coping with stress conditions, including antibiotic exposure [31,55]. Condensates can achieve this goal by shutting down vital processes so that the cell enters a dormant state, while preserving function of the involved proteins to enable rapid reactivation once the stress is over [56]. FtsZ-SlmA-SBS condensates are potentially well suited for this purpose. They would efficiently block an essential process, cytokinesis, by sequestering FtsZ inside a dynamic compartment carrying, among other factors, a strong antagonist of Z-ring assembly (SlmA), and recruiting a positive regulator (ZapA) to neutralize its action. Interestingly, persister cells tolerant to antibiotics display slow or arrested growth [56,57] linked to downregulation of GTP biosynthesis through (p)ppGpp signalling [58], a condition that would tip the balance toward FtsZ-SlmA-SBS condensation. Recovery of normal division functions under favourable growth conditions would be quite straightforward, as polymerization of FtsZ within the condensates would be triggered by the resulting higher levels of GTP, and the negative impact of SlmA would be counteracted by ZapA above a certain concentration threshold. Participation of biomolecular condensates in the mechanisms of bacterial survival to antibiotics suggests that these structures and the interactions determining their assembly may be important targets to be explored for fighting a major health threat, antimicrobial resistance. Since FtsZ and its interactions are already considered emerging targets for this purpose, aiming at the biomolecular condensates formed by this protein seems a very promising strategy.

## 4. Material and methods

Further detailed information on protein purification, FCS, fluorescence anisotropy and confocal microscopy can be found in the electronic supplementary material. The buffers mostly used across this study were crowding buffer (50 mM Tris-HCl pH 7.5, 1 mM MgCl_2_, 150 g l^−1^ dextran and, except when stated, 300 mM KCl) and solution buffer (50 mM Tris-HCl pH 7.5, 5 mM MgCl_2_ and 100 or 300 mM KCl, as specified).

### 4.1. Protein purification, labelling and DNA hybridization

FtsZ and SlmA were purified as described in [6,22,25]. ZapA was purified following, with some modifications (see electronic supplementary material, Methods), a protocol described previously [35], using a BL21(DE3) strain carrying plasmid pEJR029b. Proteins were stored in aliquots at −80°C. For the experiments, proteins were equilibrated at the specified KCl concentration in 50 mM Tris-HCl pH 7.5 and with 0–10 mM MgCl_2_.

FtsZ and SlmA were labelled at their amino groups with Alexa Fluor 488 or Alexa Fluor 647 carboxylic acid succinimidyl ester dyes (Thermo Fisher Scientific) as previously described [25,59]. ZapA was labelled with Alexa Fluor 488 carboxilic acid succinimidyl ester dye by 30 min incubation at room temperature of the protein dialysed in 50 mM HEPES pH 7.5, 300 mM KCl, 1 mM EDTA with the reactive dye, using a 1 : 7 (ZapA : dye) molar ratio. The free dye was subsequently removed using a 5 ml HiTrap Desalting column (GE Healthcare) and the labelled protein stored a −80°C. In all cases the labelling ratio, calculated from the molar absorption coefficients of the proteins and the dyes, was below 1 mole of dye per mole of protein.

Double-stranded DNA containing the SBS sequence (5′-AAGTAAGTGAGCGCTCACTTACGT-3′, bases recognized by the protein underlined [22]) was obtained by hybridization of complementary HPLC-grade oligonucleotides (IDT or Invitrogen), either unlabelled or labelled with the specified fluorophore in the 5′ terminus, as described [25].

### 4.2. Turbidity assays and determination of *c*_sat_

Condensate formation was assessed through turbidity measurements using a Varioskan Flash plate reader (Thermo Fisher Scientific, MA, USA) following a protocol described elsewhere [8]. Dextran, used as crowder, was previously dialysed and prepared as described [7]. Briefly, 125 µl of each sample were prepared in clear polystyrene, flat bottom, half-area microplates (Corning) and the absorbance at 350 nm was recorded after 30 min incubation at room temperature. In the samples containing GTP, absorbance values were taken immediately after nucleotide addition. Results are the average of at least three independent experiments ± s.d.

The concentration threshold for condensate formation, *c*_sat_, was determined from the dependence of the turbidity signal with protein concentration by fitting a linear model to the data scaling with the protein concentration (4–12 µM). The *c*_sat_ value corresponds to the x intercept.

The buffer for these experiments was crowding buffer (see above; FtsZ-SlmA-SBS condensates conditions) or 50 mM Tris-HCl, pH 7.5, 10 mM MgCl_2_ and 100 mM KCl with 200 g l^−1^ dextran (FtsZ condensates conditions).

### 4.3. Preparation of condensates in bulk solution for imaging

The bulk solutions were prepared by directly adding the protein(s) to the solution containing the crowder, and, when required, polymerization of FtsZ was triggered by direct addition of GTP. Samples with condensates were observed by confocal microscopy, except when otherwise stated, after 30 min incubation. Images were acquired with different combinations of dyes (at 0.5–1 µM depending on total protein concentrations) with equivalent results. In capture experiments, the diffusion of additional FtsZ or ZapA into the preformed labelled condensates was monitored with time by imaging the samples before and after the addition of the element labelled with a dye spectrally different from that of the condensates. The buffer for these experiments was the same as for the turbidity experiments.

### 4.4. Analytical ultracentrifugation

SE and SV experiments were performed in an Optima XLI ultracentrifuge equipped with Raleigh interference and UV-VIS absorbance optics (Beckman-Coulter), in an An-50Ti rotor using 12 mm double-sector centrepieces, at 48 Krpm (samples involving FtsZ oligomers) or 38 Krpm (samples involving FtsZ polymers) for SV and at 15 Krpm for SE. Association of ZapA was characterized following the absorbance at 230 or 280 nm at 48 Krpm. Profiles entailing Alexa 488-labelled protein were followed at 280 and 495 nm. Experiments entailing FtsZ polymers included GTP and a GTP regeneration system (RS, 2 units ml^−1^ acetate kinase and 15 mM acetyl phosphate, both from Merck) to stabilize assembled FtsZ during the measurements [60]. In all cases, experiments were conducted in solution buffer (see above) with the specified KCl concentration (100 or 300 mM). Sedimentation coefficient profiles were obtained by least square boundary modelling of SV data using the c(s) method as implemented in SEDFIT [61].

### 4.5. Fluorescence correlation spectroscopy

FCS measurements were performed on a Microtime 200 (PicoQuant) time-resolved confocal fluorescence microscope equipped with a pulsed laser diode head (LDH-P-C-485) for excitation, focused into the sample through a water immersion objective (UPLSAPO 60x Ultra-Planapochromat, NA 1.2). The fluorescence emission signal passed through a 50 µm pinhole that rejected out of focus light and was detected by a single-photon avalanche diode detector (SPAD). A 600 nm long-pass dichroic mirror and a 525/50 band-pass filter were placed before the detector. Experiments were performed and analysed essentially as previously described in ([59,62], see electronic supplementary material for additional details).

Measurements with FtsZ in its polymeric state, in the presence or absence of ZapA and SlmA-SBS complex, were conducted with 10 µM FtsZ, 2 mM GTP and the above-described enzymatic GTP regeneration system. Unless otherwise stated, 10 nM ZapA-Alexa 488, FtsZ-Alexa 488 or SBS-Alexa 488 were used as tracers, and additional unlabelled protein was added to reach the final concentrations. Measurements were performed at least in duplicate. These experiments were carried out in solution buffer with 100 or 300 mM KCl as specified.

### 4.6. Fluorescence anisotropy

Anisotropy experiments were conducted in a Spark® Multimode microplate reader (Tecan) with 485 and 535 nm excitation and emission filters, respectively, using 384 or 96 black polystyrene (non-binding surface), flat bottom microplates (Corning). For binding experiments, solutions contained ZapA-Alexa 488 as tracer (unless otherwise stated, 10 or 50 nM for ZapA alone or in the presence of FtsZ, respectively). The temperature was 26°C, unless otherwise stated. For the experiments of ZapA self-association, buffers contained 0.05 g l^−1^ BSA (Sigma; dialysed in 50 mM Tris-HCl pH 7.5, 300 mM KCl) to avoid unspecific adsorption of the protein during sample preparation. In all cases, reported values are the average of three independent replicates ± s.d. Additional details and data analysis using BIOEQS software [63] and MATLAB (ver. 7.10; MathWorks, Natick, MA, USA) can be found in the electronic supplementary material.

To monitor the time-dependent FtsZ depolymerization, the anisotropy of samples containing the protein (with 50 nM FtsZ-Alexa 488 as tracer) in the presence and absence of ZapA and/or SlmA-SBS nucleoprotein complex was recorded with time, after GTP addition. Curves representative of, at least, three independent measurements are included in the figures. The temperature was 27°C, unless otherwise indicated. All anisotropy measurements were performed in solution buffer with the specified KCl concentration, with 150 g l^−1^ dextran in figure 5*c*, or in crowding buffer (electronic supplementary material, figure S12b).

### 4.7. Microfluidics encapsulation

Microfluidic devices, constructed by conventional soft lithographic techniques, and lipid preparation were conducted as explained [37]. Encapsulation was achieved by mixing two aqueous streams in a 1 : 1 ratio prior to the droplet formation junction (electronic supplementary material, figure S13e), so final concentrations in the microdroplets are half those in the aqueous solutions. Typically, FtsZ (24 µM) was added to one of the aqueous phases, and SlmA (10 µM) and SBS (2 µM) with or without ZapA (24 µM) were added to the other. Proteins and SBS oligonucleotide labelled with Alexa 488 or Alexa 647 were used as tracers (2 µM). For the induction of FtsZ polymerization before encapsulation, the nucleotide GTP (4 mM) was included in the solution without FtsZ. The third stream supplied the mineral oil with the *E. coli* lipid mixture (20 g l^−1^; Avanti Polar Lipids). Solutions were delivered at 150 µl h^−1^ (oil phase) and 10 µl h^−1^ (aqueous phases) by automated syringe pumps (Cetoni GmbH, Germany), and production in the microfluidic chip was monitored with an Axiovert 135 fluorescence microscope (Zeiss). Microdroplets were collected and observed 30 min after production. The buffer for these experiments was the same as for the turbidity experiments.

### 4.8. Confocal microscopy

The samples in bulk solution and the collected microdroplets generated by microfluidics were visualized with a Leica TCS-SP5 inverted confocal microscope and the images analysed essentially as described [7,8]. The buffer for these experiments was the same as for the turbidity experiments. See electronic supplementary material for additional details.

## Data Availability

The data are provided in the electronic supplementary material [64].
